# Recurrent Autonomic Dysreflexia due to Chronic Aortic Dissection in an Adult Male with Cervical Spinal Cord Injury

**DOI:** 10.1100/tsw.2008.80

**Published:** 2008-06-13

**Authors:** Subramanian Vaidyanathan, Peter L. Hughes, Tun Oo, Bakul M. Soni

**Affiliations:** ^1^ Regional Spinal Injuries Centre, District General Hospital, Southport, PR8 6PN, UK; ^2^ Department of Radiology, District General Hospital, Southport, PR8 6PN, UK

**Keywords:** autonomic dysreflexia, spinal cord injury, aortic dissection

## Abstract

Autonomic dysreflexia is a hypertensive clinical emergency for persons with spinal cord injury at T-6 level or above. *Recurrent* autonomic dysreflexia is uncommon in spinal cord injury patients and is usually caused by noxious stimuli that cannot be removed promptly, e.g., somatic pain, abdominal distension. A 61-year-old man, who sustained tetraplegia at C-5 (ASIA-A) 38 years ago, was admitted with chest infection. Computerised tomography (CT) of the chest showed the ascending aorta to measure 4 cm in anteroposterior diameter; descending thoracic aorta measured 3.5 cm. No dissection was seen. Normal appearances of abdominal aorta were seen. He was treated with noninvasive ventilation, antibiotics, and diuretics. Nineteen days later, when there was sudden deterioration in his clinical condition, CT of the pulmonary angiogram was performed to rule out pulmonary embolism. This showed no pulmonary embolus, but the upper abdominal aorta showed some dissection with thrombosis of the false lumen. Blood pressure was controlled with perindopril 2 mg, once a day, doxazosin 4 mg, twice a day, and furosemide 20 mg, twice a day. Since this patient did not show clinical features of mesenteric or lower limb ischaemia, the vascular surgeon did not recommend subdiaphragmatic aortic replacement.

This patient subsequently developed recurrent episodes of autonomic dysreflexia. Each acute episode of dysreflexia was controlled by nifedipine given sublingually in doses varying from 5 to 20 mg. No inciting cause for autonomic dysreflexia could be found other than chronic aortic dissection. This patient’s medication was then changed to doxazosin 8 mg, twice a day, and sustained-release nifedipine 10 mg, twice a day, which helped to prevent recurrent autonomic dysreflexia.

Chronic aortic dissection is a very rare cause for *recurrent* autonomic dysreflexia in ageing spinal cord injury patients. When the inciting cause for dysreflexia is not amenable for treatment, recurrent dysreflexic episodes can be prevented by pharmacotherapy with an alpha-adrenergic blocking agent (doxazosin) and sustained-release nifedipine.

## INTRODUCTION

Autonomic dysreflexia is a hypertensive clinical emergency and commonly occurs in individuals with spinal cord injury at T-6 level or above. It is characterised by acute elevation of arterial blood pressure; an increase in systolic blood pressure greater than 20–30 mmHg, accompanied by intact baroreflex-mediated bradycardia, is considered a dysreflexic episode. Autonomic dysreflexia is caused by massive sympathetic discharge triggered by noxious or non-noxious stimuli below the level of the spinal cord injury. Autonomic dysreflexia is usually short lived because the triggering stimulus is dealt with promptly by health professionals, e.g., a blocked urethral catheter is changed and urinary bladder is emptied without delay. Very rarely, the noxious stimulus for autonomic dysreflexia may not be readily amenable for treatment; an example is chronic widespread pain[1].

Due to the inherent limitation of physical activity, a chronic spinal cord injury leads to an array of metabolic disorders (including insulin resistance and dyslipidemia) that can cause endothelial dysfunction, atherosclerosis, and cardiovascular disease[2]. We report a 61-year-old man with tetraplegia, who developed chronic dissection of the abdominal aorta with thrombosis of false lumen. Subsequently, this patient presented with recurrent episodes of autonomic dysreflexia and no other inciting cause could be found for the recurrent dysreflexic episodes. This is the first report of recurrent autonomic dysreflexia that was correlated with chronic dissection of the abdominal aorta.

## CASE REPORT

In 1969, this gentleman, 23 years old at the time, was driving a lorry with heavy stones in it. He tipped up the lorry to unload the stones. The stones did not fall, however; therefore, he went round to the back of the lorry and then was presumably struck by a boulder. He developed tetraplegia C-5 (ASIA-A). After undergoing rehabilitation, he lived in a care home. Over the years, he put on weight and, especially, a considerable amount of visceral adiposity. In 1998, a blood test revealed cholesterol of 5.6 mmol/L; LDL: 4.1 mmol/L and HDL: 1.0 mmol/L. In September 2004, this patient's blood pressure was 127/87 mmHg; heart rate 55 beats per minute. In June 2005, his blood pressure was 137/91 mmHg; heart rate 50 beats per minute. This patient with spinal cord injury did not suffer from chronic pain; he did not require analgesics or medicines, such as a tricyclic antidepressant, gabapentin, or pregabalin, to control neuropathic pain.

In January 2007, this patient was admitted to the spinal unit with chest pain and falling oxygen saturation. $SaO_2$ was 80% while breathing air and increased to 92% while breathing 60% oxygen. Blood pressure was 170/90 mmHg; heart rate 70 beats per minute. Coarse crepitations were present all over his chest. Computerised tomography (CT) of the chest showed the ascending aorta to measure 4 cm in anteroposterior diameter; descending thoracic aorta measured 3.5 cm. No dissection was seen. Normal appearances of the abdominal aorta were seen. He was treated with noninvasive ventilation, antibiotics, and diuretics. Nineteen days later, when there was sudden deterioration in his clinical condition, CT of the pulmonary angiogram was performed to rule out pulmonary embolism. This showed no pulmonary embolus. There was right basal atelectasis and left lower lobe collapse. Small basal pleural effusions were seen. The heart was enlarged. The upper abdominal aorta showed some dissection with thrombosis of false lumen. Blood pressure was controlled with perindopril 2 mg, once a day, doxazosin 4 mg, twice a day, and furosemide 20 mg, twice a day.

This patient was advised to avoid activities that could increase intra-abdominal pressure and, thereby, increase aortic dissection, e.g., bending forwards. Cholesterol was 5.2 mmol/L; HDL: 0.9 mmol/L; cholesterol/HDL ratio was six. This patient was prescribed atorvastatin 20 mg and aspirin 75 mg a day. He continued to have a long-term, indwelling urethral catheter.

In February 2007, this patient developed sweating, especially while sitting up. Sweating was considered to be one of the features of autonomic dysreflexia in a person with cervical spinal cord injury. This patient's bowel and bladder management were reviewed, as large bowel loaded with faeces, or a hyper-reflexic detrusor, could be triggering dysreflexic episodes. His bowel movements were satisfactory.

Since this patient had an indwelling Foley catheter, he was prescribed modified-release oxybutynin 10 mg, once a day. Oxybutynin would control bladder spasms and thereby prevent dysreflexic episodes emanating from hyper-reflexic neurogenic bladder. However, even after taking oxybutynin, this patient continued to develop sweating while sitting up. During an episode of sweating, blood pressure was 233/123 mmHg; heart rate 51 beats per minute. He was given nifedipine 5 mg sublingually; this dose of nifedipine had to be repeated three times at intervals of 5 min in order to lower blood pressure.

Concerns were raised as to whether an incorrectly placed Foley balloon might be triggering recurrent episodes of autonomic dysreflexia[3]; therefore, a cystogram was performed to check the position of the Foley catheter. The cystogram showed that the Foley balloon was located in the bladder and the catheter was draining urine satisfactorily. No inciting cause for autonomic dysreflexia could be found on detailed clinical examination. He was advised to take nifedipine 5–10 mg sublingually whenever he developed autonomic dysreflexia. This dose of nifedipine was repeated if blood pressure continued to remain high. During an episode of autonomic dysreflexia, this patient's blood pressure was persistently high despite taking a total of 20 mg nifedipine sublingually (blood pressure: 238/129 mmHg with heart rate of 64 per minute; blood pressure: 228/131 mmHg with heart rate of 69 per minute; blood pressure: 240/135 mmHg with heart rate of 66 per minute). He was then prescribed isosorbide dinitrate 0.05% (500 µg/mL), which was given intravenously, undiluted, with a syringe pump at the rate of 4 mL/h; the dose was increased, if required, at increments of 2 mL. Blood pressure decreased to 142/84 mmHg and heart rate increased to 81 per minute.

In November 2007, this patient presented to the spinal unit with a history that every time he sat up, he started sweating and this was becoming unbearable. The urethral catheter was draining correctly. He was taking Manevac granules (senna fruit: 12.4%, ispaghula: 54.2%) 10 ml after supper and had no problem with bowel movement. During an episode of sweating when sitting up, his blood pressure was 163/91 mmHg and heart rate was 51 beats per minute. Following sublingual administration of nifedipine 5 mg, blood pressure decreased to 125/66 mmHg, but after 40 min, blood pressure increased to 166/79 mmHg and heart rate was 52 beats per minute. This patient continued to develop recurrent episodes of increased blood pressure and sweating when he sitting up. In order to control recurrent episodes of autonomic dysreflexia, this patient's medication was changed to doxazosin 8 mg, twice a day, and sustained-release nifedipine 10 mg, twice a day.

CT of the abdomen was performed to look for any intra-abdominal pathology that might be generating noxious stimuli for the occurrence of recurrent autonomic dysreflexia. CT showed dissection of the abdominal aorta just below the diaphragm (Fig. 1). There was no compromise of the superior mesenteric artery or celiac axis. There was complete thrombosis of the aortic lumen just below the origin of the right renal artery. The thrombosed aortic lumen expanded progressively to the expense of the perfused aortic lumen (Fig. 2). Collateral vessels from the superior mesenteric artery were feeding the inferior mesenteric artery and further collaterals from the inferior mesenteric artery in the pelvis were feeding into small iliac vessels. Since there were no symptoms of mesenteric or lower limb ischaemia, the vascular surgeon did not recommend subdiaphragmatic aortic replacement. With a combination of sustained-release nifedipine and doxazosin, dysreflexic episodes could be prevented and this patient was discharged home after 9 days of hospitalisation. Unfortunately, he expired 4 weeks later because of chest infection.

## DISCUSSION

This tetraplegic patient with chronic aortic dissection developed recurrent episodes of autonomic dysreflexia as manifested by profuse sweating when sitting up. Neuropathic lumbar spondylolisthesis is an established cause for posture-induced autonomic dysreflexia in spinal cord injury patients[4], but CT of the lumbar spine in our patient did not show vertebral collapse or spondylolisthesis (Fig. 3), thus ruling out neuropathic lumbar spondylolisthesis as a possible cause for recurrent autonomic dysreflexia.

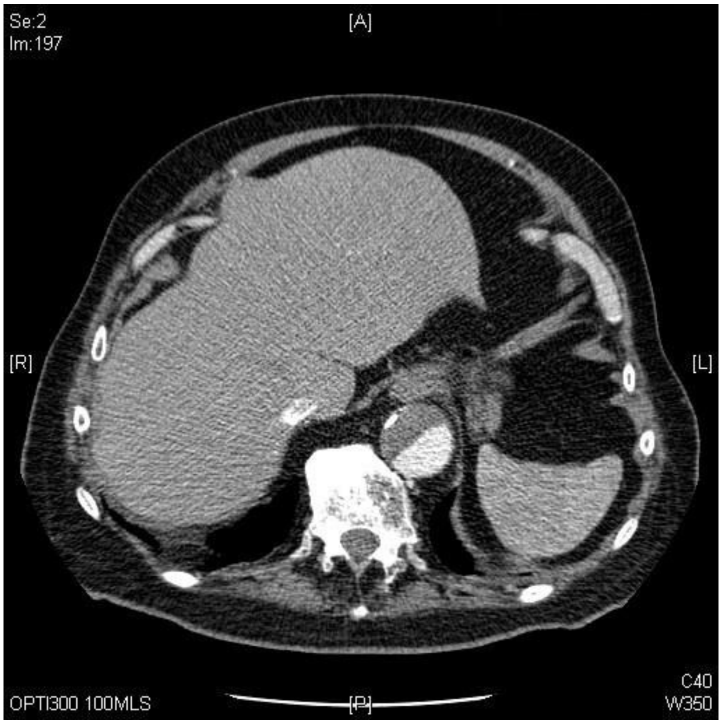

**FIGURE 1.** Contrast-enhanced CT of abdomen (axial view) shows dissection of abdominal aorta and thrombus in false lumen.

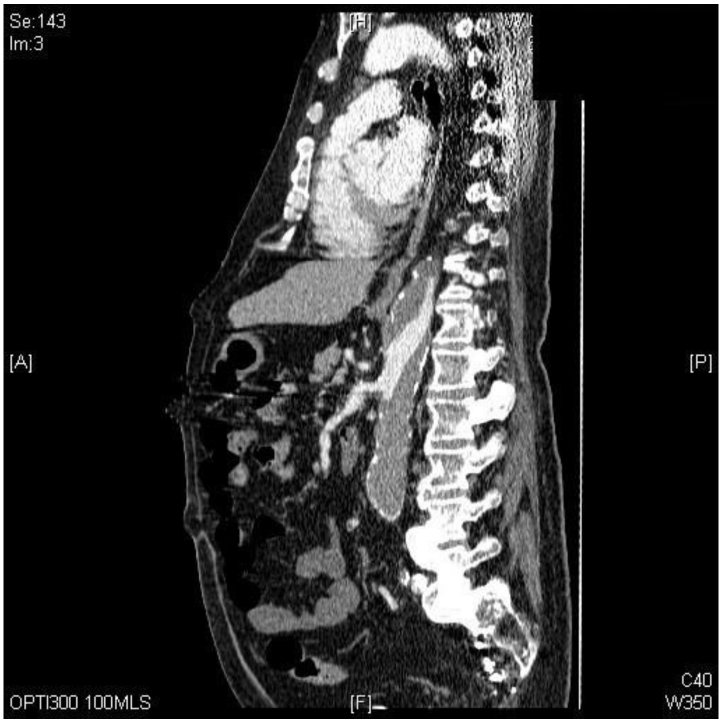

**FIGURE 2.** Contrast-enhanced CT of abdomen (sagittal reconstruction) shows dissection of abdominal aorta and thrombus in false lumen. The thrombosed aortic lumen expanded progressively to the expense of the perfused aortic lumen.

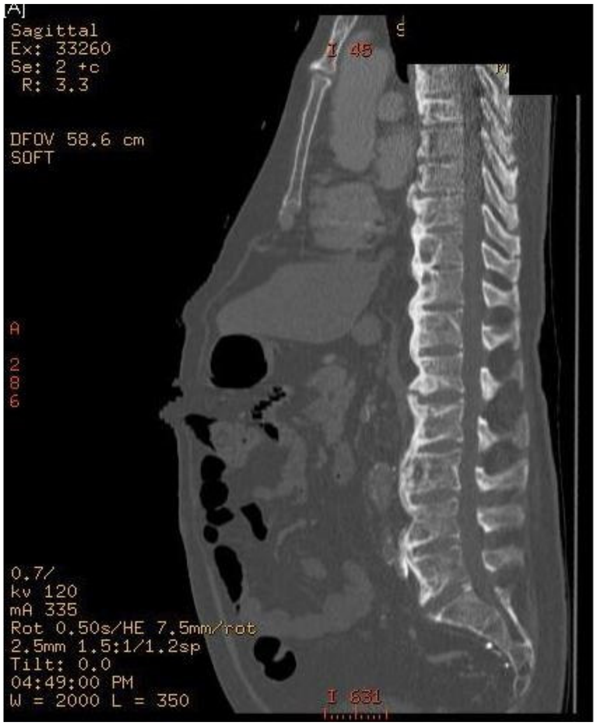

**FIGURE 3.** CT of abdomen (sagittal reconstruction) shows normal alignment of lumbar spine with no evidence of collapse of body of vertebra or spondylolisthesis.

Protracted, severe, autonomic dysreflexia has been termed "malignant", owing to the tendency of progressive worsening not usually seen with the usual clinical picture of autonomic dysreflexia, which subsides once the alleviating factor is removed. Elliott and Krassioukov[5] described three individuals in whom autonomic dysreflexia was initially triggered by ejaculation; autonomic dysreflexia continued for a period of more than 1 week. Systolic blood pressure in these individuals increased above 220 mmHg and required either acute hospitalisation or hospital assessment. Our patient with chronic aortic dissection developed recurrent episodes of autonomic dysreflexia, as manifested by increase in blood pressure greater than 180/100 mmHg on several occasions between February and November 2007. Such a clinical manifestation of chronic aortic dissection has not been reported so far.

Treatment of recurrent autonomic dysreflexia could be challenging. Elliott and Krassioukov[5] treated malignant autonomic dysreflexia in one of their three patients by administering propanolol 10 mg. We were able to prevent recurrent autonomic dysreflexia in our patient by a combination of sustained-release nifedipine 10 mg, twice daily, and doxazosin 8 mg, twice daily. In the past, we had treated 21 patients who exhibited recurrent features of autonomic dysreflexia (in the absence of an acute predisposing factor for dysreflexia, e.g., performance of an invasive procedure, such as cystoscopy, digital evacuation of bowels, or acute urinary retention) with terazosin, the dose varying from 1 to 10 mg[6]. However, the present case is the first report of a spinal cord injury patient with chronic aortic dissection, who manifested recurrent autonomic dysreflexia. This patient required not only doxazosin, which is analogous to terazosin, but also sustained-release nifedipine 10 mg, twice a day, for satisfactory control of autonomic dysreflexia.

Intrathecal chemical rhizotomy, complete sacral rhizotomy, dorsal root ganglionectomy[7], and sacral deafferentation[8] had been performed in an attempt to control autonomic dysreflexia when the noxious stimulus for autonomic dysreflexia arose from the urinary bladder, but in our patient, the inciting cause was chronic aortic dissection and, therefore, it was unlikely that any of these surgical procedures would have abated the recurrent dysreflexic episodes completely.

The guidelines produced by the Paralyzed Veterans of America/Consortium for Spinal Cord Medicine[9] recommend that if nifedipine is used, it should be in the immediate-release form. Bite-and-swallow is the preferred method of administration of nifedipine. Sublingual nifedipine administration can lead to erratic absorption. The guideline panel supported the use of an intravenous drip of sodium nitroprusside for rapid titration of blood pressure in an appropriately monitored setting. If 2% nitroglycerin ointment is used, 1 in. should be applied to the skin, above the level of spinal cord injury. Isosorbide dinitrate can be administered by intravenous infusion in glucose 5% or sodium chloride 0.9%. Isosorbide dinitrate is adsorbed to some extent by polyvinyl chloride infusion containers and, therefore, glass or polyethylene containers should be used or given via a syringe pump.

Prior to the use of nitrates, such as nitroglycerin, isosorbide dinitrate, or sodium nitroprusside, a person with spinal cord injury presenting with acute autonomic dysreflexia should be questioned regarding use of a phosphodiesterate type-5 inhibitor. If this agent has been used within the last 24 h, it is recommended that an alternative short-acting, rapid-onset, antihypertensive agent be used. Examples of agents with such characteristics are prazosin and captopril. Both drugs have an onset of action within 30 min, achieve peak serum levels within 1–3 h, and have elimination rate half-lives of 2–4 h. Our patient did not use sildenafil, tadalafil, or vardenafil for the treatment of erectile dysfunction; therefore, we were able to use isosorbide dinitrate safely.

Rarely, chronic aortic dissection may present as a febrile disease. Gorospe and associates[10] presented a case of a patient with an aortic dissection whose diagnosis was delayed because persistent fever, malaise, and night sweats dominated his clinical picture. Geppert and associates[11] reported two cases of chronic aortic dissection whose prominent features were characterised by systemic symptoms (prolonged low-grade fever, fatigue, weight loss) and biological acute phase response. Our patient did not develop fever, but clinical presentation of recurrent autonomic dysreflexia that was correlated with chronic dissection of abdominal aorta is most unusual and we are unable to find a similar case in PubMed.

Individuals with spinal cord injury have been reported to have an increased prevalence of premature cardiovascular disease. Patients with spinal cord injury were shown to have greater atherosclerotic burden than able-bodied controls[12]. Cardiovascular diseases are the most frequent cause of death among persons with spinal cord injury, and these diseases are reported to occur prematurely in the disabled compared to the able-bodied population[13]. This case illustrates the importance of the need to implement appropriate measures to *prevent* cardiovascular disease in persons with spinal cord injury. It is worth advising spinal cord injury patients on (1) healthy eating[14], combined with adequate physical activity; (2) avoidance of obesity, especially visceral adiposity; and (3) possible use of statins in ageing spinal cord injury patients, who have high cholesterol/HDL ratio. However, the authors acknowledge that physical activity is one of the most challenging parts of managing this population. There are limitations in implementing exercise activities, especially in individuals with high cervical spinal cord injury.

## CONCLUSION

Chronic aortic dissection is a very rare cause for *recurrent* autonomic dysreflexia in ageing spinal cord injury patients. When the inciting cause for dysreflexia is not amenable for treatment, recurrent dysreflexic episodes can be prevented by pharmacotherapy with an alpha-adrenergic blocking agent (doxazosin) and sustained-release nifedipine.

## References

1. Widerstrom-Noga, E., Cruz-Almeida, Y., and Krassioukov, A. (2004) Is there a relationship between chronic pain and autonomic dysreflexia in persons with cervical spinal cord injury? *J. Neurotrauma* **21(2),** 195–204.

2. Vaziri, N.D. (2007) Potential risks of nitric oxide inhibition in spinal cord injury. *J. Spinal Cord Med.* **30(1),** 3–4.
3. Vaidyanathan, S., Hughes, P.L., and Soni, B.M. (2006) A simple radiological technique for demonstration of incorrect positioning of a Foley catheter with balloon inflated in the urethra of a male spinal cord injury patient. *TheScientificWorldJOURNAL* **6,** 2445–2449.
4. Thumbikat, P., Ravichandran, G., and McClelland, M.R. (2001) Neuropathic lumbar spondylolisthesis--a rare trigger for posture induced autonomic dysreflexia. *Spinal Cord* **39(11),** 564–567.
5. Elliott, S. and Krassioukov, A. (2006) Malignant autonomic dysreflexia in spinal cord injured men. *Spinal Cord* **44(6),** 386–392.
6. Vaidyanathan, S., Soni, B.M., Sett, P., Watt, J.W., Oo, T., and Bingley, J. (1998) Pathophysiology of autonomic dysreflexia: long-term treatment with terazosin in adult and paediatric spinal cord injury patients manifesting recurrent dysreflexic episodes. *Spinal Cord* **36(11),** 761–770.
7. McGuire, E.J. and Savastano, J.A. (1984) Urodynamic findings and clinical status following vesical denervation procedures for control of incontinence. *J. Urol*. **132(1),** 87–88.
8. Kutzenberger, J. (2007) Surgical therapy of neurogenic detrusor overactivity (hyperreflexia) in paraplegic patients by sacral deafferentation and implant driven micturition by sacral anterior root stimulation: methods, indications, results, complications, and future prospects. *Acta Neurochir. Suppl.* **97(Pt 1),** 333–339.
9. Paralyzed Veterans of America/Consortium for Spinal Cord Medicine (2001) Acute management of autonomic dysreflexia: individuals with spinal cord injury presenting to health-care facilities. Paralyzed Veterans of America (PVA), Washington, D.C. http://www.guideline.gov/summary/summary.aspx?ss=15&doc_id=2964&nbr=2190
10. Gorospe, L., Sendino, A., Pacheco, R., Alonso, A., Barbado, F.J., and Vázquez, J.J. (2002) Chronic aortic dissection as a cause of fever of unknown origin. *South. Med. J.* **95(9),** 1067–1070.
11. Geppert, A.G., Mahvi, A., Hainaut, P., and Lambert, M. (1998) Chronic aortic dissection masquerading as systemic disease. *Acta Clin. Belg*. **53(1),** 19–21.
12. Orakzai, S.H., Orakzai, R.H., Ahmadi, N., Agrawal, N., Bauman, W.A., Yee, F., Adkins, R.H., Waters, R.L., and Budoff, M.J. (2007) Measurement of coronary artery calcification by electron beam computerized tomography in persons with chronic spinal cord injury: evidence for increased atherosclerotic burden. *Spinal Cord* **45(12),** 775–779.
13. Bauman, W.A. et al. (1992) Depressed serum high density lipoprotein cholesterol levels in veterans with spinal cord injury. *Paraplegia* **30,** 697–703.
14. British Heart Foundation: What is a Healthy Diet? http://www.bhf.org.uk/keeping_your_heart_healthy/healthy_eating/what_is_a_healthy_diet.aspx
